# Light-dependent expression of flg22-induced defense genes in *Arabidopsis*

**DOI:** 10.3389/fpls.2014.00531

**Published:** 2014-10-09

**Authors:** Satoshi Sano, Mayu Aoyama, Kana Nakai, Koji Shimotani, Kanako Yamasaki, Masa H. Sato, Daisuke Tojo, I. Nengah Suwastika, Hironari Nomura, Takashi Shiina

**Affiliations:** ^1^Graduate School of Life and Environmental Sciences, Kyoto Prefectural UniversityKyoto, Japan; ^2^Biology Department, Faculty of Science, Tadulako UniversityPalu, Indonesia; ^3^Department of Health and Nutrition, Gifu Women's UniversityGifu, Japan

**Keywords:** photosynthesis, flg22, defense gene, DBMIB, DCMU, retrograde signaling, salicylic acid, CAS

## Abstract

Chloroplasts have been reported to generate retrograde immune signals that activate defense gene expression in the nucleus. However, the roles of light and photosynthesis in plant immunity remain largely elusive. In this study, we evaluated the effects of light on the expression of defense genes induced by flg22, a peptide derived from bacterial flagellins which acts as a potent elicitor in plants. Whole-transcriptome analysis of flg22-treated *Arabidopsis thaliana* seedlings under light and dark conditions for 30 min revealed that a number of (30%) genes strongly induced by flg22 (>4.0) require light for their rapid expression, whereas flg22-repressed genes include a significant number of genes that are down-regulated by light. Furthermore, light is responsible for the flg22-induced accumulation of salicylic acid (SA), indicating that light is indispensable for basal defense responses in plants. To elucidate the role of photosynthesis in defense, we further examined flg22-induced defense gene expression in the presence of specific inhibitors of photosynthetic electron transport: 3-(3,4-dichlorophenyl)-1,1-dimethylurea (DCMU) and 2,5-dibromo-3-methyl-6-isopropyl-benzoquinone (DBMIB). Light-dependent expression of defense genes was largely suppressed by DBMIB, but only partially suppressed by DCMU. These findings suggest that photosynthetic electron flow plays a role in controlling the light-dependent expression of flg22-inducible defense genes.

## Introduction

Over the course of their evolution, plants have developed defense systems against a broad-spectrum of pathogens. Plant cells recognize pathogens through pattern-recognition receptors (PRRs) that recognize common features of microbial pathogens, termed pathogen-associated molecular patterns (PAMPs). The recognition of PAMPs by PRRs rapidly initiates downstream signaling events that result in the activation of an array of basal defense responses (PAMP-triggered immunity, PTI; Chisholm et al., 2006; Göhre and Robatzek, 2008). Furthermore, effector-triggered immunity (ETI) induces cell death at infection sites to enclose the spread of pathogens, a process also known as the hypersensitive reaction (HR). Plant immunity activates signal transduction pathways such as the mitogen-activated protein kinase (MAPK) phosphorylation cascades, and Ca^2+^ and reactive oxygen species (ROS) signaling pathways, which lead to transcriptional reprogramming and defense responses, including the accumulation of salicylic acid (SA), a critical signaling molecule in plant immunity. There are two distinct pathways that produce SA from chorismate in plants: the isochorismate (ICS) pathway in chloroplasts and the phenylalanine ammonia-lyase (PAL) pathway in the cytoplasm. Recently, it was demonstrated that SA is synthesized in chloroplasts via the ICS pathway, but not in the cytoplasm, in *Arabidopsis* (Fragnière et al., 2011).

PAMPs induce the expression of a specific set of defense genes, a process that is mediated by transcription factors (TFs) such as WRKYs (Rushton et al., 2010; Ishihama et al., 2011). A subset of genes activated by PAMPs is also induced by abiotic stresses such as temperature and drought. Furthermore, plant immune responses are modulated by circadian rhythms as well as abiotic stresses, including light and temperature (Hua, 2013). These facts suggest the presence of crosstalk between biotic and abiotic stress signaling pathways (Fujita et al., 2006).

Light is a fundamental factor in the control of many important biological processes during plant development and environmental responses. There is increasing evidence that light is also required for the appropriate induction of plant defense responses against pathogens (Roberts and Paul, 2006; Kangasjärvi et al., 2012). Zeier et al. (2004) demonstrated that light is responsible for accumulating SA and suppressing bacterial growth. Furthermore, several studies have shown that specific photoreceptors are involved in the regulation of plant immune responses (Griebel and Zeier, 2008; Jeong et al., 2010; Wu and Yang, 2010; Cerrudo et al., 2012). Chloroplasts may also be involved in the light-mediated control of plant immune responses. Göhre et al. (2012) reported that the flg22 peptide derived from bacterial flagellins induces down-regulation of the non-photochemical quenching of excess excitation energy (NPQ) in chloroplasts, suggesting a role for chloroplasts in plant immunity. In fact, it was recently demonstrated that the perception of PAMPs generates a transient Ca^2+^ increase in the chloroplast stroma within a few minuetes (Manzoor et al., 2012; Nomura et al., 2012). These findings suggest that PAMP signals are rapidly relayed to chloroplasts in the early stage of a plant's immune response, and support the idea that chloroplasts mediate light-dependent defense responses against infection by pathogens (Nomura et al., 2012).

Light is not only the energy source for carbon assimilation in chloroplasts, but also an important regulatory factor for chloroplast functions, such as carbon metabolism and other metabolic processes, as well as the expression of chloroplast-encoded genes. In chloroplasts, ROS are unavoidably generated with photosynthetic electron flow, which is driven by light. Singlet oxygen (^1^O_2_) is generated around photosystem II (PS II), and the superoxide anion radical (O^−^_2_) and hydrogen peroxide (H_2_O_2_) are generated around photosystem I (PS I). The ^1^O_2_ and H_2_O_2_ that are photo-produced in the chloroplast mediate retrograde signals to regulate the expression of nuclear-encoded defense genes (Kim et al., 2012; Kangasjärvi et al., 2013; Karpiński et al., 2013; Szechyńska-Hebda and Karpiński, 2013 and the hypersensitive response (Jelenska et al., 2007). CAS has been identified as a thylakoid membrane-localized Ca^2+^-binding protein that regulates cytoplasmic Ca^2+^ signals and stomatal closure (Han et al., 2003; Nomura et al., 2008; Vainonen et al., 2008; Weinl et al., 2008). We previously reported that CAS may play a role in the ^1^O_2_-mediated retrograde signaling for defense responses (Nomura et al., 2012). Based on our findings, we inferred that CAS is involved in the flg22-induced Ca^2+^ elevation in chloroplasts and in retrograde signaling from the chloroplast to nucleus to control the expression of nuclear-encoded defense genes, including SA biosynthesis genes. Excess light has been shown to activate defense-related genes, possibly through redox changes of the plastoquinone (PQ) pool (Mühlenbock et al., 2008). Furthermore, it has been suggested that the photosynthetic electron transport chain is involved in plant immune (Mateo et al., 2006; Mühlenbock et al., 2008) and stress (Jung et al., 2013) responses. However, the exact role of photosynthesis in the regulation of plant immunity remains unknown.

A large proportion of the biochemical reactions and molecular regulations occurring in chloroplasts is influenced by light. Thus, we predicted that flg22-induced defense gene expression may also be light-dependent. To elucidate the role of light and photosynthesis in flg22-induced defense gene expression, we examined the effects of light/dark conditions and photosynthesis inhibitors on the flg22-regulated expression of nuclear-encoded defense genes. We found that photosynthetic electron flow plays a key role in controlling the light-dependent expression of flg22-inducible defense genes.

## Materials and methods

### Plant materials and growth conditions

*Arabidopsis thaliana* wild-type (WT) Columbia ecotype was used in this study. Sterilized *Arabidopsis* seeds were germinated on solid agar medium consisting of 0.8% (w/v) plant tissue culture grade agar supplemented with 0.5 × Murashige and Skoog (MS) medium (Wako Chem. Co., Osaka, Japan) and grown at 22°C with 16 h light (80–100 μmol m^−2^ s^−1^)/8 h dark cycles for 2 weeks. To avoid mechanical stress when plants were treated with flg22, 2-weeks-old WT plants were floated on 0.5 × strength MS medium for 24 h before flg22 treatment. The dark plants were pre-incubated in the dark for 24 h, while the light plants were illuminated for 4 h before flg22 treatment. Both plants were treated with 1 μM flg22 for 30 min in the dark or light (80–100 μmol m^−2^ s^−1^). For treatment with photosynthesis inhibitors, plants were incubated with 5 μM 2,5-dibromo-3-methyl-6-isopropyl-benzoquinone (DBMIB) or 8 μM 3-(3,4-dichlorophenyl)-1,1-dimethylurea (DCMU) for 30 min prior to flg22 treatment (1 μM). The flg22 peptide was purchased from BIOLOGICA Co. (Nagoya, Japan). DBMIB and DCMU were purchased from Wako Chem. Co. (Osaka, Japan).

### Microarray experiments

The genome-wide microarray analyses were performed using the *Arabidopsis* V4 2 color microarray (Agilent Technologies). Total RNA was isolated from plants treated with flg22 in the light or dark for 30 min using the Qiagen RNeasy Plant Mini kit following the manufacturer's instructions. Each 200-ng total RNA sample was used to prepare Cy3- or Cy5-labeled target cRNA with the Low Input Quick Amp Labeling Kit (Agilent Technologies, USA) and used in dual color microarray hybridization with the Agilent *Arabidopsis* v4 oligo microarray slide. A dye-swap experiment was performed with two different RNA populations to eliminate the signal variation caused by the differential labeling efficiency of Cy3 and Cy5 dyes using the SuperScan microarray scanner (Agilent Technologies, USA). The microarray data were normalized by the LOWESS method using Feature Extraction software v. 10.7 (Agilent Technologies) and the expression ratios were analyzed (Non-Uniformity Outlier and Feature Population Outlier). Data with a *P*-value of >0.01 were eliminated. The genes showing a consistent expression pattern in the light or dark (>2.0 difference) are listed in sData 1.

### Microarray data analysis

The genes induced by flg22 for 30 min (Lyons et al., 2013; http://www.nature.com/srep/2013/131009/srep02866/full/srep02866.html#supplementary-information) and by illumination of the *flu* mutant (Laloi et al., 2007) were obtained from the indicated publications. Light-responsive genes in the absence of flg22 were obtained from a public database (Michael et al., 2008; http://www.ebi.ac.uk/arrayexpress/experiments/E-MEXP-1304/). Gene ontology and MapMan analysis were performed with the *Arabidopsis* Classification SuperViewer at the BAR of the University of Toronto (http://bar.utoronto.ca/ntools/cgi-bin/ntools_classification_superviewer.cgi). We compared the gene expression profiles from our microarray experiments with available expression data via the expression browser at the BAR. We also searched for overrepresented *cis*-elements in the 500-bp upstream regions of the down- and up-regulated genes in flg22-treated *cas-1* plants using the Regulatory Sequence Analysis tool (RSAT; http://rsat.ulb.ac.be/rsat/).

### qRT-PCR experiments

Plants were grown and treated with flg22 and photosynthesis inhibitors as described above in Section Plant Materials and Growth Conditions. RNA was extracted from the leaves using the RNeasy Plant Mini kit (Qiagen, USA) and cDNA was generated using SuperScript III (Invitrogen, USA). The Ct values were determined using an iCycler (Bio-Rad, USA) and analyzed with CFX Manager (Bio-Rad, USA). Primers used for qRT-PCR analyses are listed in sTable 1. Expression levels of *UBQ10* were constant under flg22 treatments. At least three independent biological replicates were performed for each sample and control. Representative results are shown as the mean ± s.e.m. of at least three technical experiments.

### SA analysis by LC/MS

SA was measured using a conventional high-performance liquid chromatography system. A total of 200 mg of seedling samples without roots was homogenized and extracted with 100% methanol containing the internal standard anisic acid. Free and glycosylated SA were separated and analyzed by liquid chromatography-tandem mass spectrometry (LC/MS/MS) (3200QTRAP, AB SCIEX, USA) with a modification of the methods described in Nomura et al. (2012).

## Results

### Light-dependent expression of flg22-induced defense genes

To identify genes rapidly responsive to flg22 whose expression is controlled by light, we performed microarray analysis of light- and dark-dependent gene expression in *Arabidopsis* seedlings treated with flg22 for 30 min. In flg22-treated plants, expressions of 3192 and 2860 genes significantly increased and decreased (more than two-fold), respectively, in the light compared to the dark control (sData 1). Under our experimental conditions, plants were illuminated for 4 h before flg22 treatment, whereas dark control plants were kept in the dark. Thus, in order to exclude light-responsive gene sets that are not regulated by flg22, we identified genes that are induced (1612 genes, >2.0) or repressed (1496 genes, <0.5) by 4 h illumination in the absence of flg22 from the public database (Michael et al., 2008). As a result, 616 of 1612 light-induced genes and 699 of 1496 light-repressed genes overlap with the light-dependent and -repressed genes identified in the flg22-treated plants based on our microarray data, respectively. Thus, we removed these genes from our microarray data, and obtained 2576 light-dependent genes and 2161 light-repressed genes in plants treated with flg22 for 30 min. The resultant datasets were used for further analyses.

In order to focus on genes that are rapidly regulated by flg22 in a light-dependent manner, we compared the light-dependent and -repressed genes described above with genes rapidly responsive to flg22 that were identified by Lyons et al. (2013). They showed that flg22 induced the expression of 3579 genes within 30 min (>2.0), whereas it repressed the expression of 4159 genes (<0.5) (Lyons et al., 2013). We identified a large number of genes that are induced by flg22 in a light-dependent manner (sData 2); 536 (14.9%) of 3579 flg22-induced genes overlapped significantly with the light-dependent genes (named flg22-induced light-dependent genes), but less with light-repressed genes [239 (6.7%) genes, named flg22-induced light-repressed genes] (Table 1). We further named the genes that are induced by flg22 but light-insensitive as flg22-induced light-independent genes. As shown in Table 2, the top 25 flg22-induced genes include a large number of light-dependent genes, but not flg22-repressed genes. These results suggest that light is a critical signal for the activation of defense gene expression. Gene ontology analysis revealed that stress-responsive and signal transduction-related genes were overrepresented among the flg22-induced light-dependent genes, but not in the flg22-induced light-repressed genes (sFigure 1). Furthermore, MapMan analysis revealed that functional categories related to stress, signaling, hormone metabolism, and tetrapyrrole synthesis were significantly enriched in the flg22-induced light-dependent genes (sTable 2). It should be noted that the light-induced genes include a number of genes involved in SA biosynthesis, such as *EDS1*, *PAD4*, *SAG101*, *EDS5*, and *PAL1*, but include fewer TF-related genes compared with the light-independent genes.

**Table 1:** **The number of genes up- and down-regulated by flg22 and light**.

	Flg22-responsive genes*		Light-responsive genes**		**Overlap genes**	*P*-value^***^
Flg22-induced light-dependent genes	3579	(flg22-up)	2576	(light-dependent)	536	8.790e-24
Flg22-induced light-repressed genes	3579	(flg22-up)	2161	(light-repressed)	239	1.084e-05
Flg22-repressed light-dependent genes	4159	(flg22-down)	2576	(light-dependent)	359	2.599e-04
Flg22-repressed light-repressed genes	4159	(flg22-down)	2161	(light-repressed)	370	0.15

The light-dependent and -repressed genes identified in this study (^**^) were compared with flg22 rapidly responsive genes identified by Lyons et al. (2013) (^*^). P-value (

*** ) was calculated using the hypergeometric probability formula.

**Table 2:** **Top 25 genes induced and repressed by flg22 in 30 min**.

**AGI code**	**Description**	flg22-up^*^ fold change	Light-up^**^ fold change
**flg22-INDUCED GENES WITHIN 30 MIN**			
AT5G24110	WRKY30;_transcription_factor	604.357	4.08
AT1G06135	similar_to_unknown_protein_(TAIR:AT2G31345.1)	229.879	2.32
AT3G02840	immediate_early_fungal_elicitor_family_protein	220.241	–
AT4G14450	Identical_to_Uncharacterized_protein_At4g14450	206.208	–
AT1G22810	AP2_domain-containing_transcription_factor,_putative	179.432	–
ATIG19210	AP2_domain-containing_transcription_factor,_putative	175.500	–
AT5G47850	protein_kinase,_putative	172.464	2.25
AT2G31345	similar_to_unknown_protein_(TAIR:AT1G06135.1)	164.362	–
AT3G12910	transcription_factor	153.511	3.08
AT1G06137	similar_to_unknown_protein_(TAIR:AT1G06135.1)	150.495	–
AT2G37430	zinc_finger_(C2H2_type)_family_protein_(ZAT11)	149.453	3.22
AT1G72520	lipoxygenase,_putative	134.598	2.79
AT5G11140	similar_to_unknown_protein_(TAIR:AT4G38560.1)	130.349	–
AT4G18540	similar_to_unknown_protein_product_(GB:CAO48082.1)	129.095	4.79
ATIG07160	protein_phosphatase_2C,_putative	114.130	2.85
ATIG56240	ATPP2-B13_(Phloem_protein_2-B13)	106.016	4.08
AT5G64905	PROPEP3_(Elicitor_peptide_3_precursor)	96.240	2.2
AT1G56250	ATPP2-BI4_(Phloem_protein_2-BI4)	93.958	4.98
AT4G34410	AP2_domain-containing_transcription_factor,_putative	93.865	–
AT4G31950	CYP82C3_(cytochrome_P450)	93.089	6.73
AT5G05300	similar_to_unknown_protein_(TAIR:AT3G10930.1)	85.834	2.16
AT4G11470	protein_kinase_family_protein	83.563	3.75
AT4G11070	WRKY41;_transcription_factor	80.292	–
AT4G19520	disease_resistance_protein_(TIR-NBS-LRR_class)	80.044	2.16
AT5G42380	CML37/CML39;_calcium_ion_binding	71.672	3.03
**flg22-REPRESSED GENES WITHIN 30 MIN**			
AT3G42550	aspartyl_protease_family_protein	0.009	–
AT5G06500	AGL96;_DNA_binding_/_transcription_factor	0.015	–
AT2G05350	unknown_protein	0.020	–
AT4G05095	similar_to_unknown_protein (TAIR:AT4G04650.1)	0.022	–
AT4G30074	LCR19_(Low-molecular-weight_cysteine-rich_19)	0.025	–
AT5G59310	LTP4_(LIPID_TRANSFER_PROTEIN_4);_libid_bin	0.025	–
AT1G60500	dynamic_family_protein	0.027	–
AT5G24440	CID13_(CTC-Interacting_Domain_13);_RNA_bindir	0.028	–
AT3G42060	myosin_heavy_chain-related	0.031	–
AT1G66855	similar_to_glycosyl_hydrolase_family_protein_17	0.032	–
AT4G05071	unknown_protein	0.035	–
AT4G28170	similar_to_unknown_protein (TAIR:AT1G11120.1)	0.036	–
AT1G54775	Encodes_a_Plant_thionin_family_protein	0.040	–
AT5G33390	glycine-rich_protein	0.044	–
AT5G42957	similar_to_unknown_protein (TAIR:AT5G42955.1)	0.047	–
AT1G27990	similar_to_unknown_protein (TAIR:AT5G52420.1)	0.050	–
AT3G44755	similar_to_unknown_protein (TAIR:AT3G46360.1)	0.051	–
AT4G29200	beta_galactosidase	0.052	–
AT2G15590	similar_to_unknown_protein (TAIR:AT4G33985.1)	0.056	–
AT5G28560	unknown_protein	0.060	–
AT4G27415	unknown_protein	0.061	–
AT1G34280	unknown_protein	0.063	–
AT3G59460	similar_to_F-box_family_protein_(TAIR:AT3G6004)	0.068	–
AT1G70944	unknown_protein	0.069	–
AT2G36190	ATCWINV4	0.070	–

* flg22-induced (upper) and -repressed (lower) genes identified by microarray analysis by Lyons et al. (2013).

** Fold change represents ratios of mean mRNA abundance in the light to mean mRNA abundance in the dark control. Data are representative for two independent experiments (P < 0.01).

“–” indicates that genes were not induced nor repressed more than two times by light.

Among the 4159 genes suppressed by flg22 within 30 min, 359 were dependent on light (named flg22-repressed light-dependent genes) and 370 genes were repressed by light (named flg22-repressed light-repressed genes). The genes repressed by flg22 and light-insensitive are named flg22-repressed light-independent genes. The genes rapidly suppressed by flg22 include significantly fewer genes involved in stress responses irrespective of light treatment (sFigure 1). Interestingly, the flg22-repressed light-dependent genes include a large number of genes involved with electron transport or energy, structural molecule activity, and chloroplasts; transcription factor TF activity genes were significantly overrepresented among the flg22-repressed light-repressed genes.

**Figure 1:**
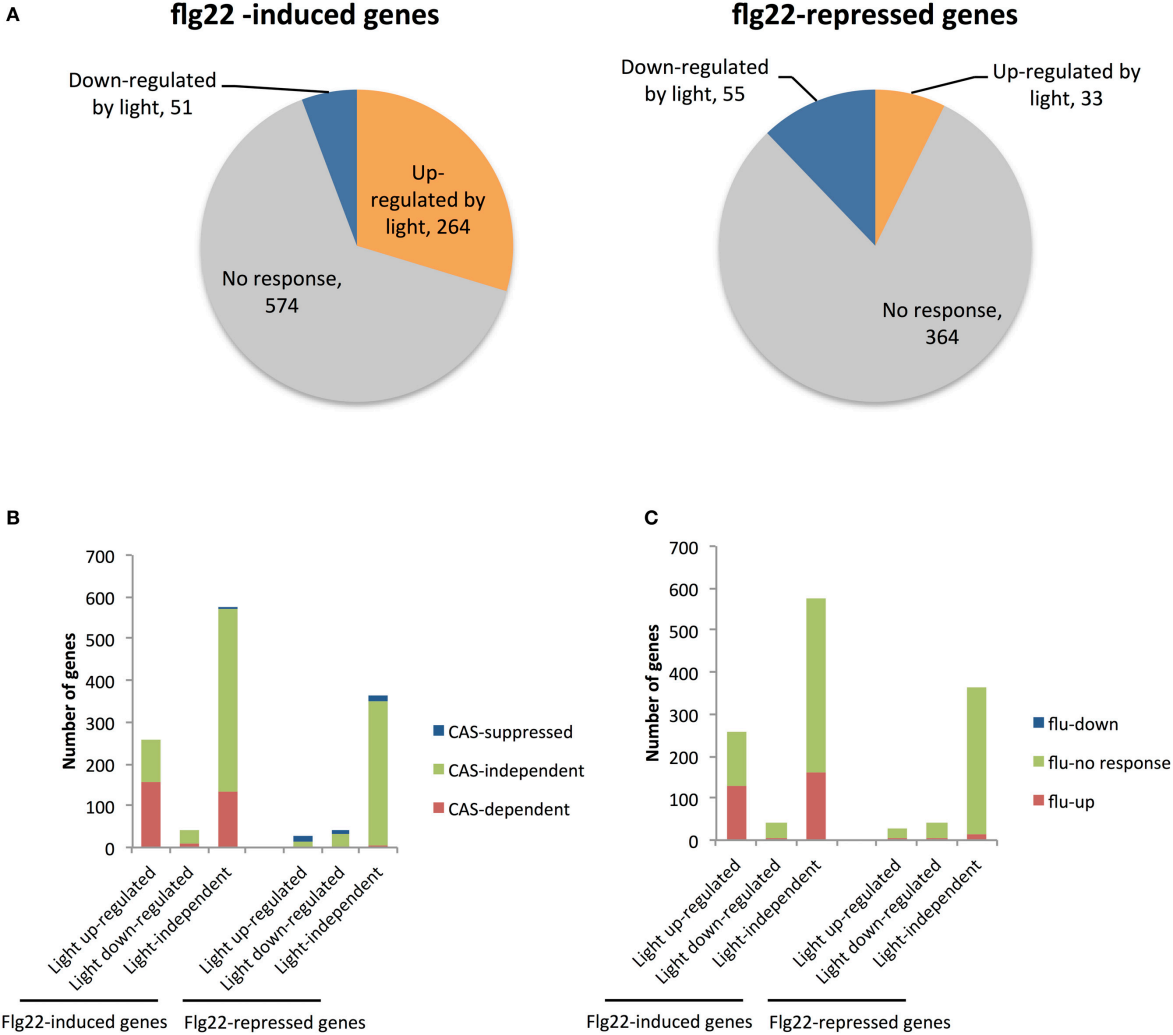
**Microarray analysis of genes up- and down-regulated by light in flg22-treated seedlings. (A)** The number of genes up- and down-regulated by light among sets of genes previously identified as strongly up- (>4.0) or down-regulated (<0.25) by flg22 within 30 min (Lyons et al., 2013). **(B)** The number of genes previously shown to be CAS-dependent (red) or CAS-suppressed (blue) or CAS-independent (green) (Nomura et al., 2012) among genes that are up- (left) and down-regulated (right) by flg22 treatment. **(C)** The number of genes previously shown to be induced in illuminated *flu* mutants (red) or suppressed (blue) (Laloi et al., 2007) among genes that are up- (left) and down-regulated (right) by flg22 treatment. Microarray analysis was performed with seedlings treated with flg22 for 30 min under light or dark conditions.

Table 2 shows that 16 of 25 genes (64%) that were markedly induced by flg22 after 30 min were also dependent on light, suggesting that light plays a critical role in the strong induction of flg22-induced genes. Thus, we further analyzed genes that are strongly (>4.0) induced by flg22. We identified 889 flg22-induced (>4.0) and 452 flg22-repressed (<0.25) genes after 30-min treatment with flg22. A comparison of these genes with the light-regulated genes identified in this study revealed that markedly flg22-induced genes include a larger number of light-dependent genes. Approximately 30% of the strongly induced genes (264 genes) were up-regulated in the light, but only 4.9% of those genes (51 genes) were down-regulated by light (Figure 1A), indicating that light plays a more significant role in the expression of genes that are largely induced by flg22.

CAS is a thylakoid membrane-localized Ca^2+^-binding protein (Han et al., 2003; Nomura et al., 2008; Vainonen et al., 2008; Weinl et al., 2008). We previously demonstrated that CAS mediates retrograde chloroplast signals to regulate flg22-induced nuclear gene expression. We identified 1235 genes that are induced to a lesser extent by flg22 in CAS knockout *cas-1* mutants than in WT plants (CAS-dependent genes), as well as 687 genes up-regulated in *cas-1* plants (CAS-repressed genes). Here, we found that the CAS-dependent genes also overlapped significantly with the flg22-induced light-dependent genes. The rapidly flg22-induced light-dependent genes include 60.5% CAS-dependent genes, whereas the flg22-induced light-independent genes contain only 23.3% CAS-dependent genes (Figure 1B). On the other hand, the rapidly flg22-induced light down-regulated genes include only one CAS-suppressed gene (Figure 1B). In contrast, the flg22-repressed genes include very few CAS-dependent genes, irrespective of light treatment. These results suggest that chloroplasts and, specifically, CAS are involved in the light-mediated control of flg22-induced nuclear defense gene expression. It has been suggested that CAS is involved in ^1^O_2_ mediated retrograde signaling, facilitating chloroplast-mediated transcriptional reprogramming during plant immune responses. As shown in Figure 1C, 127 of 258 rapidly flg22-induced light-dependent genes overlapped significantly with 1565 ^1^O_2_-responsive genes induced in illuminated *flu* mutants (fold change >3.0) (Laloi et al., 2007), suggesting that ^1^O_2_ signaling plays a role in the light-dependent activation of flg22-induced genes.

### *Cis*-element sequences overrepresented in the light-dependent and -independent flg22-induced genes

**Figure 2:**
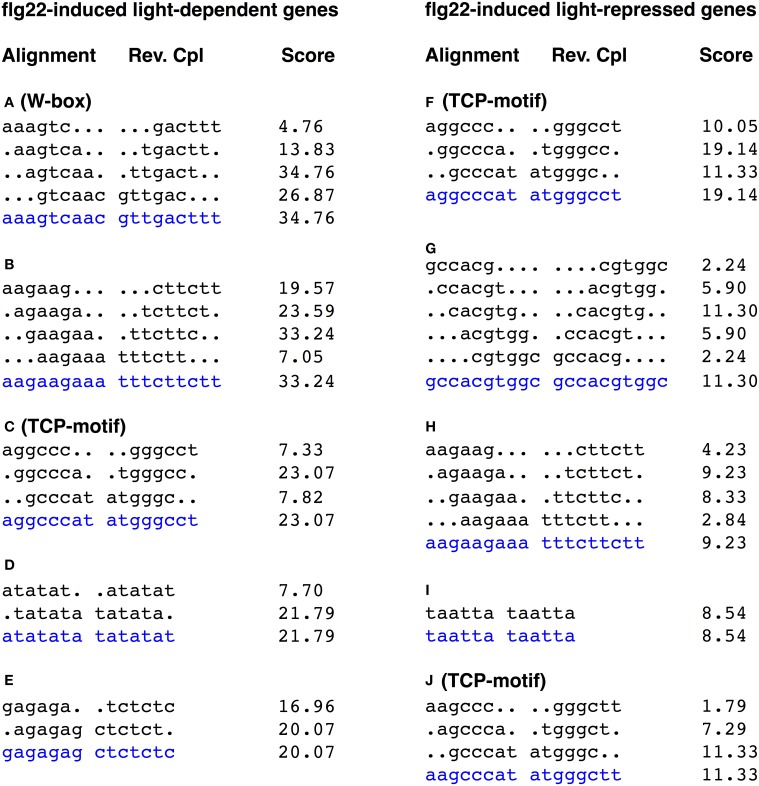
**Alignments of hexamer sequences overrepresented in the promoters of genes up- and down-regulated by light in flg22-treated seedlings**. The 500 bp upstream regions of 536 flg22 induced light-dependent genes and 239 flg22 induced light-repressed genes were subject to promoter motif analysis using the RSAT. The consensus is taken as the most common base pair in that position, and shown by blue characters. W-box was significantly overrepresented in the promoters of light-dependent genes, whereas TCP-motif was overrepresented in the promoters of both light-dependent and -independent genes. The score is calculated by the oligo analysis tool by default and is equivalent to log10 of the E-value.

We searched for overrepresented 6-bp motifs within the 500-bp upstream region of the predicted translation start sites of the flg22-induced (>2.0) and -repressed (<0.5) genes using a motif discovery tool (RSAT, http://www.rsat.eu/). Several short sequences were significantly overrepresented in promoter regions of rapidly flg22-induced and -repressed genes (Tables S3, S4). The alignment of hexamers identified two known consensus sequences overrepresented in the promoters of genes rapidly induced by flg22 treatment. The most highly represented motifs in the promoters (500-bp upstream regions) of the light-independent (*P*-value, 2e-102) and light-repressed (*P*-value, 3.5e-23) flg22-induced genes were GGCCCA (Figure 2, sTable 3), which are part of the TCP TF-binding motifs (GGNCCCAC or GGNCCC) (Li et al., 2005). The TCP-binding motif sequence GGCCCA is also overrepresented in the light-dependent genes, suggesting that TCP is unlikely to be involved in the light-mediated response of flg22-induced genes. Furthermore, GGCCCA sequences were frequently present in the promoters of the flg22-repressed genes irrespective of light treatment.

The W-box motif (TTGACC/T) was significantly overrepresented in the promoters of the flg22-induced light-dependent genes (agtcaa; *P*-value, 8.4e-39, gtcaac; *P*-value, 6.4e-31). In contrast, the W-box was less represented in flg22-induced genes repressed by light (Figure 2). The W-box is the binding motif for WRKY family TFs, which regulate biotic and abiotic stress responses (Rushton et al., 2010). Furthermore, the W-box was not represented in the promoters of flg22-repressed genes irrespective of light treatment. On the other hand, no well-known light-responsive *cis*-elements, such as GT-elements (GR(T/A)AA(T/A)), G-box elements (CACGTG), or I-box elements (GATAA), were overrepresented in the light-dependent flg22-induced genes. These results suggest that WRKY TFs are involved in the rapid light-dependent expression of flg22-induced defense genes.

### qRT-PCR of light-dependent expression of flg22-induced genes

**Figure 3:**
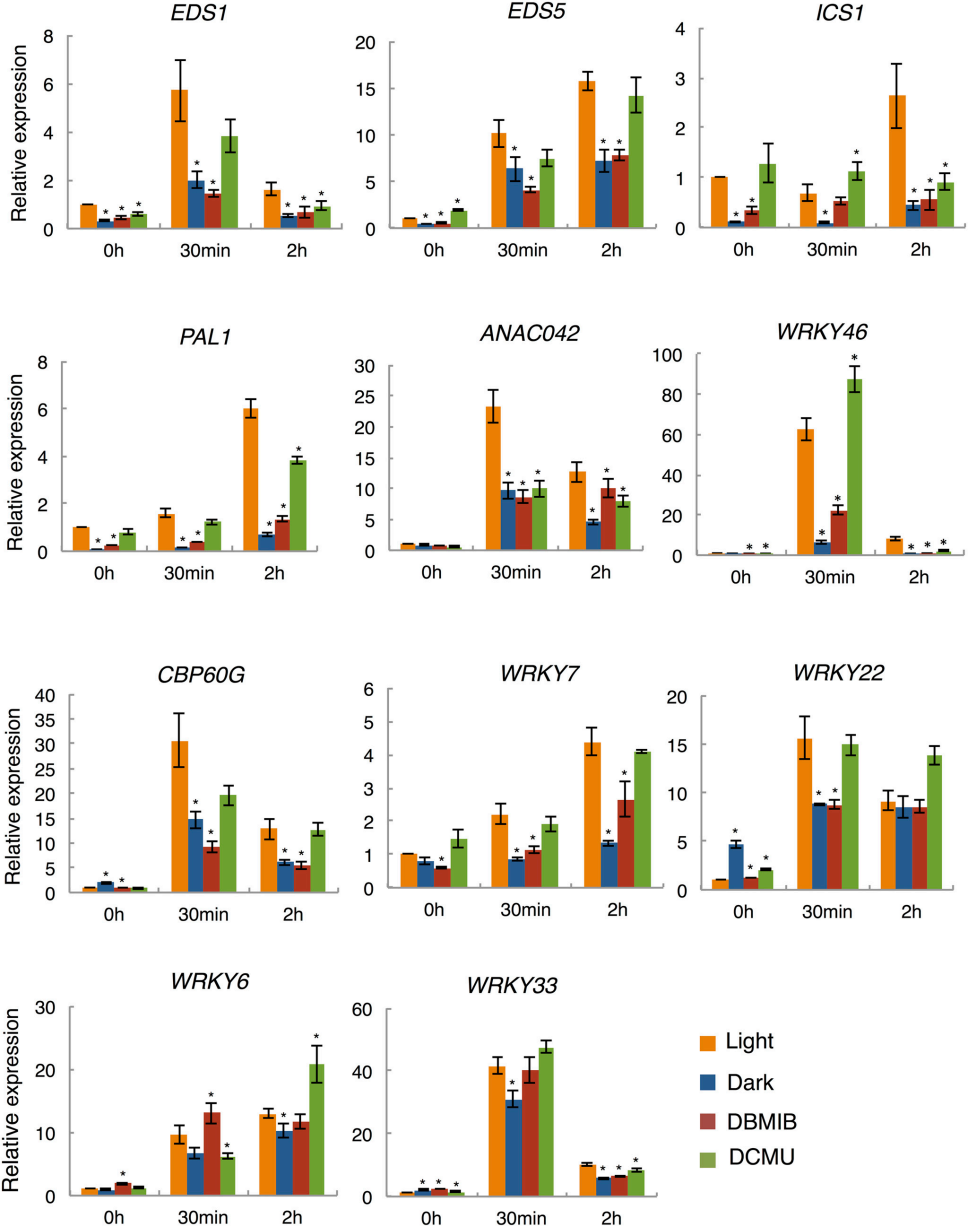
**qRT-PCR analysis of flg22-induced gene expression in the dark and light, and in the presence of photosynthesis inhibitors**. Plants were treated with 1 μM flg22 in the light (orange) or dark (blue) for 30 min and 2 h, respectively. As indicated, plants were also pretreated with DBMIB (5 μM; red) or DCMU (8 μM; green) for 30 min before flg22 treatment. *UBQ10* was used as an internal standard. Results shown are mean + s.e.m. from triplicate technical replicates from one of three representative experiments with similar results. *P*-values for qRT-PCR data were calculated using *t*-tests and are indicated by ^*^p < 0.005.

SA is a key signaling molecule in plant immune responses. We found that the expression of several key genes responsible for SA accumulation were up-regulated by light in the presence of flg22. Thus, we selected four genes involved in SA biosynthesis (*EDS1*, *EDS5*, *ICS1*, and *PAL1*) and seven TF genes that act mainly in the defense response network (*ANAC042*, *CBP60g*, *WRKY6*, *7*, *22*, *33*, and *46*) as targets for quantitative reverse transcription-PCR (qRT-PCR). According to the microarray data, the flg22-induced expression of the four SA biosynthesis genes (*EDS1*, × 2.1; *EDS5*, × 5.79; *ICS1*, × 3.61; *PAL1*, × 12.33) and two of the TF genes (*WRKY46*, × 3.37; *ANAC042*, × 2.57) was dependent on light, whereas that of the other five TF genes was not. In order to examine the effects of light on the expression of genes that are induced slowly in response to flg22 treatment, we added plants treated with flg22 for 2 h. As shown in Figure 3, the expression of all selected genes was up-regulated by flg22 either transiently (*EDS1*, *ANAC042*, *CBP60g*, *WRKY22*, *33*, and *46*) or gradually (*EDS5*, *ICS1*, *PAL1*, *WRKY6*, and *7*). As expected, qRT-PCR analysis revealed that the expression of *EDS1* (× 2.84), *ICS1* (× 7.78), *PAL1* (× 10.67), *ANAC042* (× 2.41), and *WRKY46* (× 9.33) was largely dependent on light in the *Arabidopsis* plants treated with flg22 for 30 min. These data are consistent with the microarray data. Although expression of *EDS5* was slightly dependent on light based on qRT-PCR analysis (× 1.61), there was a significant difference between light- and dark-treated samples in microarray analysis. On the other hand, flg22-induced expression of *CBP60g*, *WRKY7*, and *22* was reduced by half in the dark compared to the light in qRT-PCR experiments (× 2.08, × 2.59, and × 1.77, respectively), whereas their expression was not significantly up-regulated by light in the microarray data. Expression of genes that are induced gradually by flg22, such as *EDS5*, *ICS1*, *PAL1*, and *WRKY7*, was more significantly dependent on light after 2 h. Furthermore, it should be noted that *EDS1*, *EDS5*, *ICS1*, and *PAL1*, which are involved in SA biosynthesis, showed reduced expression in the dark even before flg22 treatment, whereas the TF-related genes did not. In contrast, the expression levels of two other genes (*WRKY6* and *33*) were not markedly decreased in the dark, confirming the microarray data.

### Effect of electron transport inhibitors on flg22-induced gene expression

In order to examine the involvement of photosynthesis in the light-dependent defense gene expression, we analyzed the expression of flg22-induced defense genes in the presence of two specific inhibitors of photosynthetic electron transport. DCMU and DBMIB inhibit electron transport from the PS II complex to the PQ pool and from the PQ pool to the cytochrome *b*_6_/*f* complex, respectively (Trebst, 2007). *Arabidopsis* seedlings were pretreated with these inhibitors for 30 min before flg22 treatment. Electron transport rates measured using a PAM chlorophyll fluorometer were suppressed by both inhibitors to less than 10% of those in non-treated plants within 30 min (sFigure 2). Importantly, the light-dependent expression of all nine flg22-induced genes examined was significantly suppressed by DBMIB (Figure 3) to levels similar to those observed in the dark after 30 min or 2 h of flg22 treatment. However, flg22-induced gene expression was not significantly suppressed or was only partially suppressed by DCMU. In contrast, *WRKY6* and *33*, which exhibited light-independent expression, were not significantly suppressed by DBMIB or DCMU, indicating that electron transport inhibitors do not affect the light-independent expression of flg22-induced defense genes. These results suggest that the photosynthetic electron flow plays a key role in regulating the light-dependent expression of flg22-induced defense genes.

### Effects of light on flg22-induced SA accumulation

**Figure 4:**
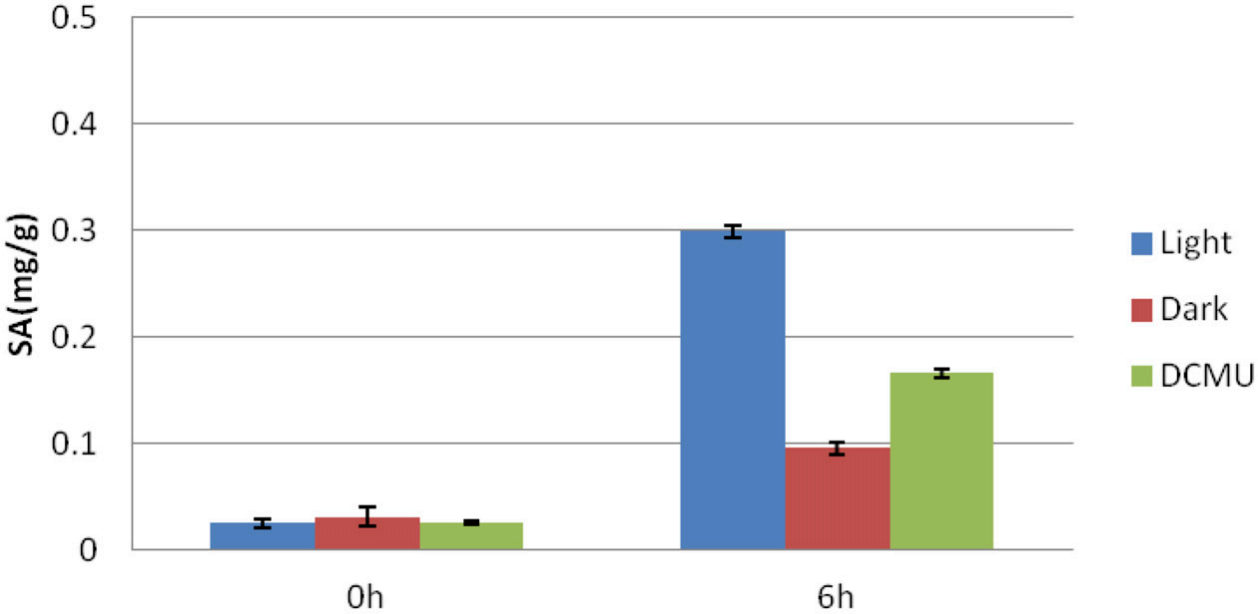
**Light-dependent salicylic acid (SA) accumulation**. Flg22-induced SA accumulation was measured in leaves in the light or dark. As indicated, some plants were treated with DCMU (8 μM) for 30 min before flg22 treatment. Bars indicate the standard error of the mean (s.e.m.).

We found that the flg22-induced expression of a set of genes involved in SA biosynthesis (*EDS1*, *ICS1*, *EDS5*, and *PAL1*) and regulation (CBP60g, WRKY7, and 46; Kim et al., 2006; Zhang et al., 2010; van Verk et al., 2011) was dependent on light (Figure 2). Therefore, to elucidate the role of light in flg22-induced SA biosynthesis, we investigated free SA accumulation in the leaves of *Arabidopsis* seedlings treated with flg22 under light and dark conditions (Figure 4). Upon flg22 treatment, *Arabidopsis* accumulated less SA in the dark than in the light. These results indicate that light is involved in the activation of SA biosynthesis by flg22.

## Discussion

It has been suggested that light is necessary for robust and precise immune responses in plants (Roden and Ingle, 2009; Wang et al., 2011). Several reports have suggested the involvement of photoreceptors, such as phytochromes (Griebel and Zeier, 2008) and cryptochromes (Jeong et al., 2010; Wu and Yang, 2010), in the plant immune system. It has also been indicated that photosynthesis is involved in the activation of SA biosynthesis, *PR1* gene expression, and HR against infection by pathogens (Jelenska et al., 2007; Kangasjärvi et al., 2012; Kim et al., 2012). We previously demonstrated that the chloroplast Ca^2+^-binding protein CAS plays a role in flg22-induced immune responses through retrograde signals originating in chloroplasts to regulate defense gene expression in the nucleus (Nomura et al., 2012). To learn more about the roles of chloroplasts and photosynthesis in PAMP-induced immunity, we investigated the effects of light and photosynthesis inhibitors on bacterial flagellin peptide flg22-induced expression of nuclear-encoded defense genes in *Arabidopsis.*

Microarray analysis revealed that a number of the rapidly flg22-induced genes are dependent on light, but not the flg22-repressed genes. These results suggest that light is required for the activation of gene expression induced by flg22. Furthermore, we examined the effects of photosynthesis inhibitors on flg22-induced gene expression in the light. The flg22-induced expression of all light-induced genes examined (*EDS1*, *EDS5*, *ICS1*, *ANAC042*, *PAL1*, *CBP60g*, *WRKY7, 22*, and *46*) was significantly suppressed by DBMIB, and the expression of *ICS1* and *ANAC042* was partially suppressed by DCMU. However, these photosynthesis inhibitors did not affect flg22-induced expression of light-independent genes (*WRKY6* and *33*). These results suggest that photosynthesis mediates the light-dependent expression of flg22-induced genes. DCMU and DBMIB inhibit the flow of electrons between PSII and the PQ pool, and between the PQ pool and PSI, respectively. In fact, both inhibitors significantly reduced the electron transport activity between PSII and PSI (sFigure 3), suggesting that the intersystem electron flow between PSII and PSI may be involved in the light-dependent regulation of flg22-induced gene expression.

It should be noted that DBMIB showed more significant effects on light-dependent gene expression induced by flg22 than DCMU. DBMIB reduced the expression of all eight light-dependent defense genes examined to the levels found in dark-adapted plants, while DCMU treatment did not significantly change the expression of defense genes (by more than two-fold) except for two genes, *ANAC042* and *ICS1*. DCMU and DBMIB have opposite effects on the redox state of the PQ pool: the PQ pool is oxidized by DCMU treatment and reduced by DBMIB treatment. Thus, the redox state of the PQ pool may be important as a signal for light-dependent flg22-induced defense gene expression in plant immunity. These findings suggest that modification of the redox state plays a critical role in the generation of chloroplast-derived signals to control nuclear-encoded defense and stress-responsive genes. On the other hand, it is known that DBMIB inhibits not only photosynthetic electron transport in chloroplasts, but also mitochondrial electron transport. Although we used a low concentration of DBMIB, it is possible that it partially affects mitochondrial functions.

Previous reports demonstrated that reduction of the PQ pool by DBMIB and excess light promoted the expression of immune genes. Furthermore, recent transcription analysis of DBMIB-treated plants revealed that PQ pool reduction promoted the expression of 798 stress-responsive genes (Jung et al., 2013). Thus, it has been suggested that the PQ redox state triggers the induction of immunity- and stress-related genes. In accordance with these findings, DBMIB-induced genes were significantly overrepresented among the flg22-induced light-dependent genes (>4.0) (28.0%), but less in the group of flg22-induced genes repressed by light and light–independent genes (16.0%) (sFigure 3). These results suggest that PQ-pool redox signaling is also involved in the light-dependent expression of flg22-induced genes. However, the light-dependent expression of flg22-induced genes was largely suppressed by DBMIB (Figure 3). A mechanism linking the flg22-induced signaling and PQ-pool redox signaling remains elusive. Further studies are needed to explore the discrepancy regarding the effects of DBMIB effects on immunity-related gene expression in the presence and absence of flg22, which may shed lights on the role of PQ-pool redox in retrograde signaling to control nuclear-encoded immunity- and stress-related genes.

Perturbation of photosynthetic electron flow promotes the generation of ROS, including ^1^O_2_ in PS II, and O^−^_2_ and H_2_O_2_ in PS I. ^1^O_2_ and H_2_O_2_ may be involved in the retrograde signals to activate nuclear-encoded defense gene expression (Kangasjärvi et al., 2013) and the HR (Kim et al., 2012). Thus, ROS are candidate retrograde signals that mediate the photosynthesis-dependent regulation of nuclear-encoded defense genes. Photosynthesis inhibitors and dark conditions may suppress the electron flow-dependent ROS immune signals and subsequent defense gene expression. It is suggested that PAMPs somehow perturb the photosynthetic electron flow that leads to the generation of chloroplast-derived ROS immune signals. Previously, we demonstrated that PAMP signals are rapidly transmitted to chloroplasts to generate a transient increase in Ca^2+^ in chloroplasts (Nomura et al., 2012). Furthermore, our previous work implicated CAS in the generation of ^1^O_2_-mediated signals to activate the flg22-induced expression of several defense genes. Recently, it was also reported that PAMPs cause a rapid decrease in NPQ after 30 min (Manzoor et al., 2012). Thus, chloroplasts may be able to quickly recognize PAMP signals, leading to changes in photosynthesis.

SA biosynthesis induced by pathogenic infection is dependent on light (Zeier et al., 2004); consistent with this, we showed that flg22-induced accumulation of SA is also dependent on light. SA biosynthesis in plants involves two distinct pathways: the ICS and the PAL pathways. Both pathways originate from chorismate, which is the end-product of the shikimate pathway (Dempsey et al., 2011). We found that light-induced genes activated by flg22 include a number of genes involved in SA biosynthesis, including *EDS1*, *PAD4*, *SAG101*, *EDS5*, *PAL1*, and *PAL2*. qRT-PCR analysis revealed that the flg22-induced expression of *EDS1*, *ICS1*, *EDS5*, and *PAL1* is suppressed by DBMIB. Light is also required for the flg22-induced expression of two TFs, *CBP60g* (Zhang et al., 2010; Wang et al., 2011) and *WRKY46* (van Verk et al., 2011), which are involved in the activation of SA biosynthesis genes. Furthermore, it should be noted that light is responsible for the expression of genes involved in SA biosynthesis even prior to flg22-treatment. Light may be involved in the priming of SA biosynthesis genes. It is known that SA accumulation is elevated in the light (Mateo et al., 2006). Thus, flg22-induced SA accumulation in the light may be partially due to the direct photosynthesis-mediated activation of SA accumulation in chloroplasts. Taken together, these results suggest that light activates the expression of SA biosynthesis genes through photosynthesis-mediated immune signals, leading to SA accumulation.

We found that W-box sequences are significantly enriched in the promoters of the light-dependent flg22-induced genes. The W-box is the binding motif for WRKY family TFs (Rushton et al., 2010). The expression of more than 70% of WRKY gene family members in *Arabidopsis* is responsive to pathogenic infection and SA treatment (Dong et al., 2003). These findings suggest that WRKY TFs play an important role in the transcription of flg22-induced defense genes in the light. Interestingly, the light-dependent flg22-induced genes (>4.0) include just three WRKY TF genes (*WRKY30*, *46*, and *53*), while 12 WRKY TF genes (*WRKY6*, *11*, *22*, *26*, *33*, *40*, *41*, *55*, *62*, and *70*) were identified among the light-independent or -repressed flg22-induced genes. Further analyses of these three WRKY TFs may shed light on the photosynthesis-mediated immune signals.

Contrastingly, the TCP-binding motif sequences are significantly overrepresented in the promoters of most flg22-regulated genes, except for light- and flg22-repressed genes. TCP family TFs are involved in the transcriptional regulation of genes controlling the cell cycle, growth, development, circadian clock, and jasmonic acid biosynthesis (Trémousaygue et al., 2003; Li et al., 2005; Welchen and Gonzalez, 2005, 2006; Schommer et al., 2008; Hervé et al., 2009; Pruneda-Paz et al., 2009). TCP TFs may also be involved in Ca^2+^-dependent transcriptional regulation in *Arabidopsis* (Whalley et al., 2011). Furthermore, the GGCCCA and AGCCCA motifs are also similar to a FORC^A^ promoter element (T/ATGGGC) (Evrard et al., 2009). FORC^A^-mediated promoter activity is induced by SA under constant light exposure, whereas SA does not activate the FORC^A^-mediated promoter under constant darkness (Evrard et al., 2009). The further characterization of TCP TFs and FORC^A^ promoter elements may provide insight into the light-mediated control of flg22-repressed genes.

In summary, this study revealed that both the up- and down-regulation of defense-related genes by flg22 is dependent on light to a large extent, and suggested that photosynthesis plays a role in the light-dependent regulation of flg22-responsive genes. It is also suggested that chloroplasts produce light-dependent retrograde signals to regulate flg22-induced nuclear gene expression. Alternatively, photosynthesis may indirectly influence defense responses. Our findings further suggest that ROS and the redox state of the PQ pool are involved in this light-dependent chloroplast-mediated immune signaling. However, the molecular mechanisms linking photosynthesis and defense gene expression remain largely elusive. Further experiments are needed to clarify light-dependent retrograde chloroplast-to-nucleus signaling, which optimizes pathogen-induced defense responses in a fluctuating light environment.

### Conflict of interest statement

The authors declare that the research was conducted in the absence of any commercial or financial relationships that could be construed as a potential conflict of interest.
