# Supplementary material for: Light-dependent expression of flg22-induced defense genes in Arabidopsis
Source: Front Plant Sci. 2014 Oct 9;5:531. doi: 10.3389/fpls.2014.00531 (PMC4191550; doi:10.3389/fpls.2014.00531)
Supplement: Supplementary file 1 [file Table1.DOCX]

sTable 1 List of primer sequences used for RT-PCR analysis in this study.

| *EDS1* | Forward | CTCAATGACCTTGGAGTGAGC |
| --- | --- | --- |
|  | Reverse | TCTTCCTCTAATGCAGCTTGAA |
| *ICS1* | Forward | GCGTCGTTCGGTTACAGG |
|  | Reverse | ACAGCGAGGCTGAATCTCAT |
| *EDS5* | Forward | TGGGAGTTATCGGAACAGCG |
|  | Reverse | CGAGGGATACTGTCATGGGC |
| *PAL1* | Forward | ATTAACGGGGCACACAAGAG |
|  | Reverse | GTCTCCGCCGCATAACATAG |
| *CBP60G* | Forward | GAGCTCGTAACTTAACATTCAAGAAA |
|  | Reverse | CTTATCATGTTCTCCATCTGAATCAT |
| *WRKY6* | Forward | GCAACAGCAACAACAGAACAA |
|  | Reverse | TGCCTTGGTACTATCGTCTCC |
| *WRKY7* | Forward | CCATTGCTCCAAGAAAAGGA |
|  | Reverse | TGGTATATCAGCCATTTTGCTACT |
| *WRKY22* | Forward | GATCATCTAGCGGTGGGAGA |
|  | Reverse | CCACCGTATCCGACAAAGC |
| *WRKY33* | Forward | GGGAAACCCAAATCCAAGA |
|  | Reverse | GTTTCCCTTCGTAGGTTGTGA |
| *WRKY46* | Forward | GAAAATGGCTCTATTGATGATGG |
|  | Reverse | TCGATGCGTGCATCTGTAA |
| *Anac042* | Forward | TACCTCTGCTTGTTGAGCTG |
|  | Reverse | AGGCACCAAAACCGATTGGA |
| *UBQ10* | Forward | GGCCTTGTATAATCCCTGATGAATAAG |
|  | Reverse | AAAGAGATAACAGGAACGGAAACATAGT |
