# Supplementary material for: Light-dependent expression of flg22-induced defense genes in Arabidopsis
Source: Front Plant Sci. 2014 Oct 9;5:531. doi: 10.3389/fpls.2014.00531 (PMC4191550; doi:10.3389/fpls.2014.00531)
Supplement: Supplementary file 2 [file Table2.DOCX]

sTable 2 Classification of light-dependent and independent genes that are induced quickly by flg22

Light-dependent Light-independent

Normed Normed

to Freq. StdDev p-value to Freq. StdDev p-value

Biodegradation of Xenobiotics 0 0 0.638 0 0 0.820

C1-metabolism 0 0 0.527 3.54 2.486 0.214 Co-factor and vitamine metabolism 2.33 1.315 0.098 0 0 0.563

DNA 0.26 0.067 **1.04E-10** 0.09 0.062 **2.511e-08**

N-metabolism 2.42 2.132 0.277 0 0 0.832

OPP 4.05 2.771 0.074 4.57 3.653 0.177

PS 0.91 0.541 0.224 0 0 0.232

RNA 0.76 0.107 0.013 1.9 0.289 **2.398e-05**

S-assimilation 0 0 0.812 0 0 0.912

TCA / org transformation 1.59 0.958 0.227 0 0 0.571

amino acid metabolism 0.96 0.455 0.197 2.18 0.975 0.075

cell 1.89 0.358 **1.02E-03** 0.85 0.346 0.166

cell wall 0.8 0.257 0.125 1.81 0.687 0.053

development 1.27 0.289 0.064 1.07 0.494 0.160

fermentation 0 0 0.799 0 0 0.906

gluconeogenesis / glyoxylate cycle 0 0 0.812 21.81 12.004 **3.577e-03**

glycolysis 1.59 0.866 0.227 0 0 0.571

hormone metabolism 1.74 0.47 0.014 1.31 0.592 0.150

lipid metabolism 1.02 0.391 0.151 0.99 0.63 0.226

major CHO metabolism 1.24 0.755 0.262 1.4 1.038 0.352

metal handling 0.75 0.685 0.355 1.7 1.157 0.328

micro RNA, natural antisense etc 0 0 **5.99E-04** 0.3 0.22 0.125

minor CHO metabolism 0.5 0.416 0.275 3.43 1.744 **0.046**

misc 1.46 0.201 **5.26E-03** 0.98 0.275 0.122

mitochondrial electron transport 0 0 0.089 0.93 0.655 0.369

/ ATP synthesis

not assigned 0.74 0.055 **2.00E-06** 0.85 0.081 **0.014**

nucleotide metabolism 1.74 0.692 0.091 2.36 1.303 0.096

polyamine metabolism 0 0 0.774 0 0 0.893

protein 0.8 0.095 **8.86E-03** 0.84 0.156 **0.049**

redox 0.89 0.49 0.222 2.02 1.183 0.124

secondary metabolism 1.13 0.394 0.133 2.55 0.822 **9.504e-03**

signalling 3.37 0.334 **7.65E-20** 1.76 0.386 **8.706e-03**

stress 2.4 0.365 **2.10E-08** 0.92 0.301 0.139

tetrapyrrole synthesis 5.24 2.506 **6.10E-03** 0 0 0.712

transport 1.41 0.29 **0.023** 0.83 0.338 0.146

P-values<0.05 are printed bold.
