# Supplementary material for: Light-dependent expression of flg22-induced defense genes in Arabidopsis
Source: Front Plant Sci. 2014 Oct 9;5:531. doi: 10.3389/fpls.2014.00531 (PMC4191550; doi:10.3389/fpls.2014.00531)
Supplement: Supplementary file 3 [file Table3.DOCX]

sTable 3 Promoter motif of upstream sequences of flg22-induced genes

Seq Identifier Obs. Exp. *P* Value ratio

(Light-dependent genes)

agtcaa agtcaa|ttgact 439 219.99 8.4e-39 2.00 W-box

gaagaa gaagaa|ttcttc 538 294.37 2.7e-37 1.83

gtcaac gtcaac|gttgac 254 111.70 6.4e-31 2.27 W-box

agaaga agaaga|tcttct 502 296.65 1.2e-27 1.69

ggccca ggccca|tgggcc 121 37.70 4.1e-27 3.21 TCP-motif

tatata tatata|tatata 432 249.49 7.7e-26 1.73

agagag agagag|ctctct 350 193.70 4.1e-24 1.81

aagaag aagaag|cttctt 473 137.44 1.3e-23 1.64

gagaga gagaga|tctctc 365 214.31 5.2e-21 1.70

ggtcaa ggtcaa|ttgacc 247 130.99 1.1e-19 1.89 W-box

(Light-repressed genes)

ggccca ggccca|tgggcc 78 19.75 3.5e-23 3.95 TCP-motif

atgggc atgggc|gcccat 76 26.25 2.2e-15 2.90

cacgtg cacgtg|cacgtg 51 13.23 2.4e-15 3.86

aggccc aggccc|gggcct 52 14.77 4.2e-14 3.52 TCP-motif

agaaga agaaga|tcttct 211 122.66 2.8e-13 1.72

taatta taatta|taatta 149 78.92 1.4e-12 1.89

gaagaa gaagaa|ttcttc 205 120.98 2.2e-12 1.69

agccca agccca|tgggct 73 30.04 2.5e-11 2.43 TCP-motif

aataat aataat|attatt 328 225.10 7.6e-11 1.46

acgtgg acgtgg|ccacgt 63 26.01 6e-10 2.42

(Light-independent genes)

ggccca ggccca|tgggcc 402 109.49 2e-102 3.67 TCP-motif

agaaga agaaga|tcttct 1496 820.53 2.3e-99 1.82

gaagaa gaagaa|ttcttc 1432 811.02 2.4e-86 1.77

aagaag aagaag|cttctt 1291 772.91 5.4e-65 1.67

taatta taatta|taatta 943 523.24 3e-61 1.80

agagag agagag|ctctct 1026 584.93 3.7e-61 1.75

gagaga gagaga|tctctc 1094 644.24 1.4e-58 1.70

tatata tatata|tatata 1148 690.19 3.6e-57 1.66

aggccc aggccc|gggcct 246 80.27 7.8e-50 3.06 TCP-motif

cttctc cttctc|gagaag 773 451.24 3.5e-43 1.71
