# Supplementary material for: Light-dependent expression of flg22-induced defense genes in Arabidopsis
Source: Front Plant Sci. 2014 Oct 9;5:531. doi: 10.3389/fpls.2014.00531 (PMC4191550; doi:10.3389/fpls.2014.00531)
Supplement: Supplementary file 4 [file Table4.DOCX]

sTable 4 Promoter motif of upstream sequences of flg22-repressed genes

Seq Identifier Obs. Exp. *P* Value ratio

(Light-dependent genes)

ggccca ggccca|tgggcc 130 33.43 8.3e-37 3.89 TCP-motif

agaaga agaaga|tcttct 358 190.54 1.9e-27 1.88

aggccc aggccc|gggcct 85 22.15 2.6e-24 3.84 TCP-motif

gaagaa gaagaa|ttcttc 338 188.01 5e-23 1.80

atgggc atgggc|gcccat 115 42.95 7.4e-20 2.68 TCP-motif

agccca agccca|tgggct 123 48.12 1.4e-19 2.56 TCP-motif

cacgtg cacgtg|cacgtg 65 18.21 1.7e-17 3.57

aagaag aagaag|cttctt 296 177.46 2.8e-16 1.67

gcccaa gcccaa|ttgggc 124 56.95 1.1e-14 2.18

aagaaa aagaaa|tttctt 630 468.02 6e-13 1.35

(Light-repressed genes)

aataat aataat|attatt 636 414.85 4.2e-24 1.53

agagag agagag|ctctct 253 129.46 5.3e-22 1.95

agaaga agaaga|tcttct 322 180.30 1.3e-21 1.79

aagaag aagaag|cttctt 304 168.66 4.8e-21 1.80

cacgtg cacgtg|cacgtg 73 19.76 3.1e-20 3.69

tatata tatata|tatata 367 235.47 1.3e-15 1.56

gaagaa gaagaa|ttcttc 281 168.42 1.5e-15 1.67

ggccca ggccca|tgggcc 71 25.21 6.6e-14 2.82 TCP-motif

aagaaa aagaaa|tttctt 644 474.01 7e-14 1.36

gagaga gagaga|tctctc 240 143.42 1.2e-13 1.67

(Light-independent genes)

ggccca ggccca|tgggcc 838 223.67 2e-216 3.75 TCP-motif

agaaga agaaga|tcttct 2848 1611.61 5e-170 1.77

gaagaa gaagaa|ttcttc 2775 1581.49 5e-162 1.75

aagaag aagaag|cttctt 2627 1514.55 8e-148 1.73

tatata tatata|tatata 2801 1713.45 4e-128 1.63

aggccc aggccc|gggcct 524 159.43 9e-115 3.29 TCP-motif

taatta taatta|taatta 2049 1218.86 3e-104 1.68

agccca agccca|tgggct 845 362.25 1e-103 2.33 TCP-motif

atgggc atgggc|gcccat 771 324.09 1.8e-98 2.38 TCP-motif

agagag agagag|ctctct 1837 1085.24 7.1e-96 1.69
