# Supplementary material for: Light-dependent expression of flg22-induced defense genes in Arabidopsis
Source: Front Plant Sci. 2014 Oct 9;5:531. doi: 10.3389/fpls.2014.00531 (PMC4191550; doi:10.3389/fpls.2014.00531)
Supplement: Supplementary file 5 [file Presentation1.PPTX]

## Slide 1
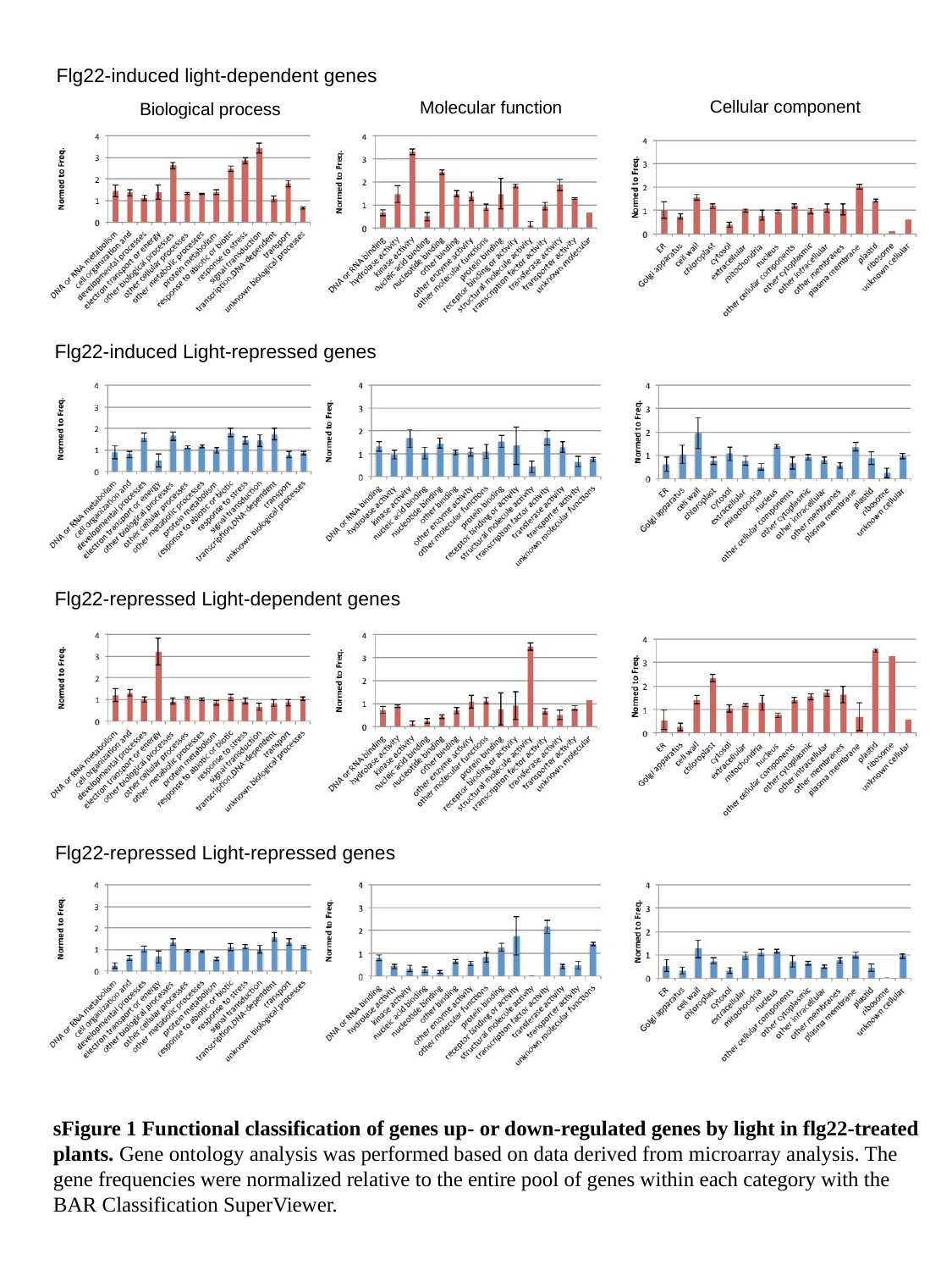

Flg22-induced light-dependent genes
Cellular component
Molecular function
Biological process
Flg22-induced Light-repressed genes
Flg22-repressed Light-dependent genes
Flg22-repressed Light-repressed genes
sFigure 1 Functional classification of genes up- or down-regulated genes by light in flg22-treated plants. Gene ontology analysis was performed based on data derived from microarray analysis. The gene frequencies were normalized relative to the entire pool of genes within each category with the BAR Classification SuperViewer.

## Slide 2
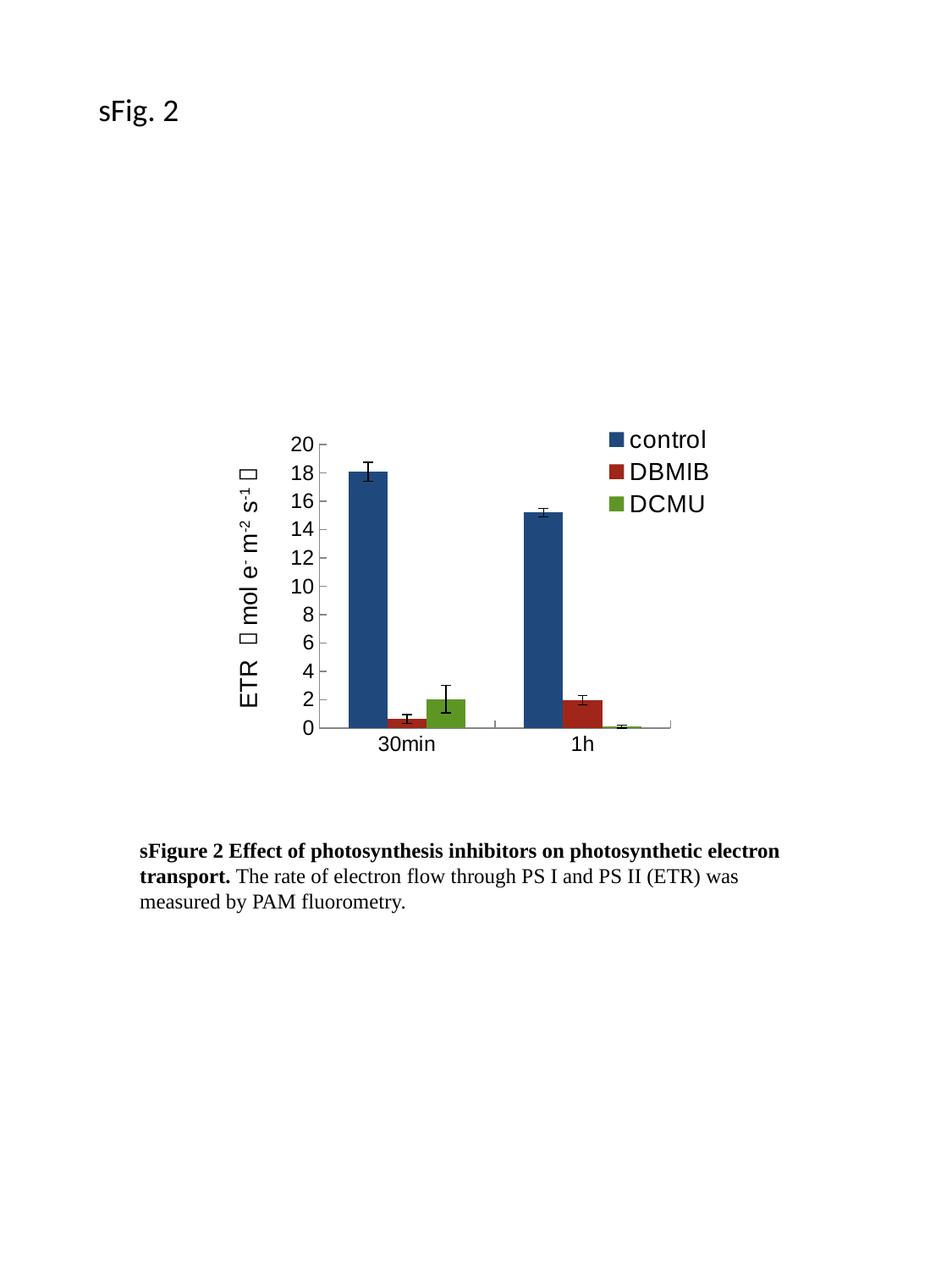

sFig. 2
### Chart
| Category | control | DBMIB | DCMU |
|---|---|---|---|
| 30min | 18.06666666666667 | 0.633333333333335 | 2.033333333333333 |
| 1h | 15.2 | 1.966666666666668 | 0.1 |ETR （mol e- m-2 s-1）
sFigure 2 Effect of photosynthesis inhibitors on photosynthetic electron transport. The rate of electron flow through PS I and PS II (ETR) was measured by PAM fluorometry.

## Slide 3
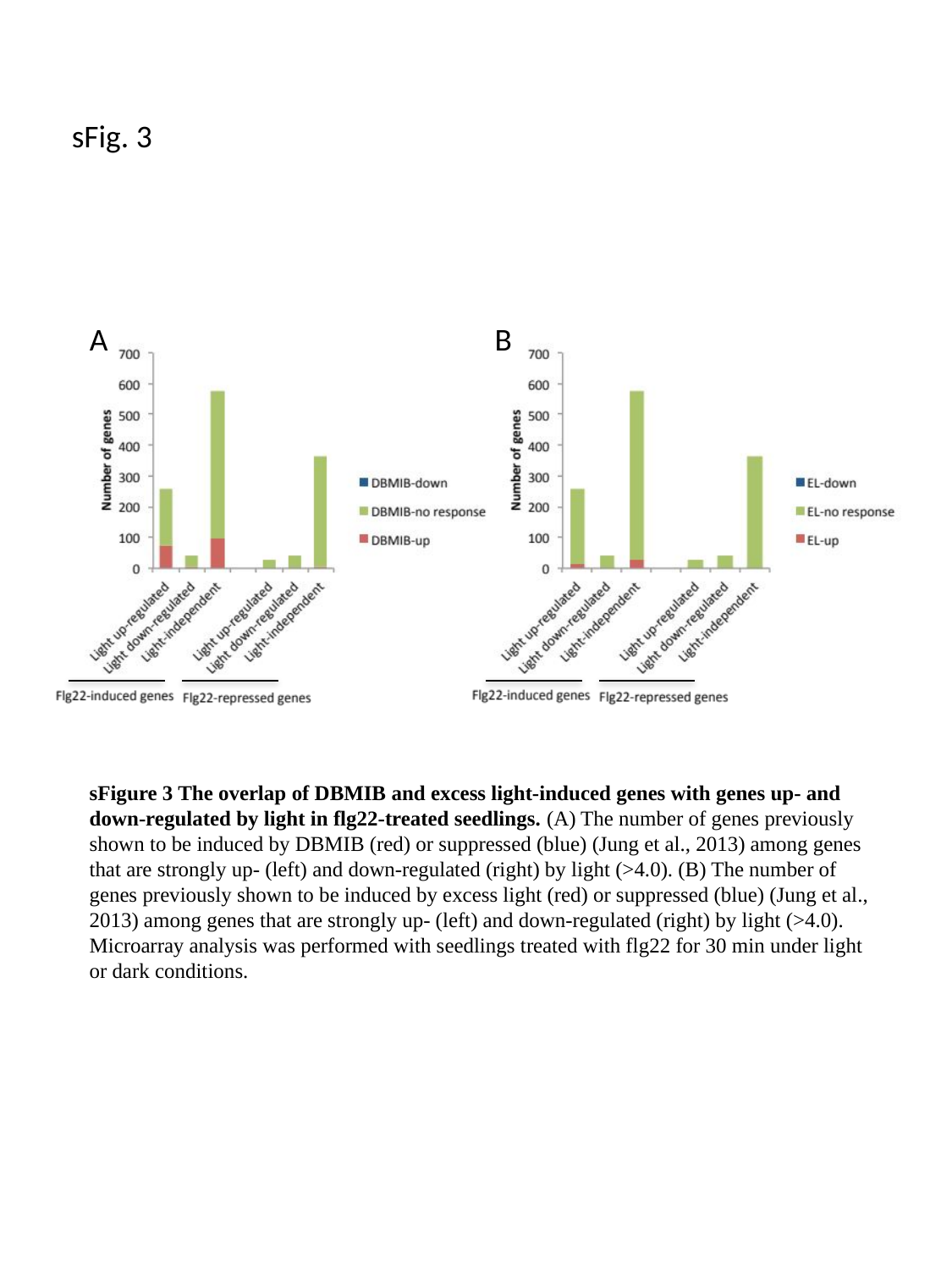

sFig. 3
A
B
sFigure 3 The overlap of DBMIB and excess light-induced genes with genes up- and down-regulated by light in flg22-treated seedlings. (A) The number of genes previously shown to be induced by DBMIB (red) or suppressed (blue) (Jung et al., 2013) among genes that are strongly up- (left) and down-regulated (right) by light (>4.0). (B) The number of genes previously shown to be induced by excess light (red) or suppressed (blue) (Jung et al., 2013) among genes that are strongly up- (left) and down-regulated (right) by light (>4.0). Microarray analysis was performed with seedlings treated with flg22 for 30 min under light or dark conditions.
